# The Hepatocyte Growth Factor/c-Met Antagonist, Divalinal-Angiotensin IV, Blocks the Acquisition of Methamphetamine Dependent Conditioned Place Preference in Rats

**DOI:** 10.3390/brainsci2030298

**Published:** 2012-08-20

**Authors:** John W. Wright, Wendy L. Wilson, Vanessa Wakeling, Alan S. Boydstun, Audrey Jensen, Leen Kawas, Joseph W. Harding

**Affiliations:** 1 Department of Psychology, Washington State University, Pullman, WA 99164-4820, USA; Email: vanessa.wakeling@gmail.com (V.W.); audreyjensen3@gmail.com (A.J.); harding@vetmed.wsu.edu (J.W.H.); 2 Department of Veterinary and Comparative Anatomy, Pharmacology, and Physiology, Washington State University, Pullman, WA 99164-6520, USA; 3 Department of Psychology, Dickinson State University, Dickinson, ND 58601, USA; Email: wendy.i.wilson@dickinsonstate.edu; 4 L-3 Communications, Link Simulation and Training, Wright Patterson Air Force Base, OH 45433-7955, USA; Email: alanboydstun@gmail.com; 5 Program in Pharmacology and Toxicology, Washington State University, Pullman, WA 99164-6510, USA; Email: kawas@wsu.edu

**Keywords:** methamphetamine, conditioned place preference, divalinal-angiotensin IV, AT_4_ receptor subtype, hepatocyte growth factor, c-Met receptor

## Abstract

The use of methamphetamine (MA) is increasing in the U.S. and elsewhere around the world. MA’s capacity to cause addiction significantly exceeds other psychostimulant drugs, and its use negatively impacts learning and memory. Recently, attempts have been made to interfere with the presumed mechanism(s) underlying the establishment of drug-induced memory consolidation. The majority of these studies have employed matrix metalloproteinase (MMP) inhibitors to disrupt MMP-induced extracellular matrix molecule dependent synaptic reconfiguration, or GABA receptor agonists. The present investigation utilized an angiotensin IV (AngIV) analogue, Divalinal-AngIV (divalinal), to disrupt acquisition of MA-induced dependence in rats as measured using the conditioned place preference paradigm. Results indicate that both acute and chronic intracerebroventricular infusion of divalinal prior to each daily subcutaneous injection of MA prevented acquisition. However, divalinal was unable to prevent MA-induced reinstatement after prior acquisition followed by extinction trials. These results indicate that prevention of MA dependence can be accomplished by blockade of the brain AT_4_ receptor subtype. On the other hand, once MA-induced memory consolidation is in place divalinal appears to be ineffective. Mechanistic studies indicated that divalinal is a potent inhibitor of the hepatocyte growth factor (HGF)/c-Met receptor system, and thus it appears that a functional HGF/c-Met system is required for the acquisition of MA-mediated conditioned place preference.

## 1. Introduction

D-Methamphetamine (MA) has become a major drug of abuse in the U.S., Canada, Mexico, and Asia [1,2]. This psychostimulant acts via the release of biogenic amines [3] with addictive effects further facilitated by blockade of presynaptic reuptake (reviewed in [4,5]), and decreased expression of membrane transporters [6]. MA also increases cytosolic levels of monoamines by inhibiting monoamine oxidase activity [7]; while increasing the activity of the dopamine synthesizing enzyme tyrosine hydroxylase [8], a key synthetic enzyme. These alterations result in a major increase in the synaptic levels of dopamine, norepinephrine, and serotonin leading to substantial synaptic remodeling, alternations in synaptic efficiency, and ultimately drug dependence. Recent strategies to curb MA dependence have focused on the inhibition of matrix metalloproteinase (MMP) in order to disrupt MMP-induced extracellular matrix molecule dependent synaptic reconfiguration [9,10], or GABA_B_ receptor agonists [11,12,13]. However, the intensity of MA dependence and the resulting persistence of drug abuse have prompted a search for novel ways to discourage the addictive process. 

It has been known for some time that the use of MA negatively impacts learning and memory [14,15,16,17]. These findings have encouraged the hypothesis that drug addiction and memory consolidation may share common neural plasticity mechanisms [18,19]. Thus, acquisition of environmental preference cues during the addiction process may be a form of learning and as such should be constrained by the same mechanisms that underlie normal learning and memory consolidation (reviewed in [20,21,22,23,24,25,26,27]). 

Several years ago our laboratory discovered a new angiotensin receptor subtype, the AT_4_ [28]. This receptor protein is heavily distributed in the neocortex, hippocampus, nucleus basalis of Meynert, ventral tegmental area and nucleus accumbens. Hippocampal AT_4_ receptors are important in learning and memory consolidation that mediates spatial and associative learning (reviewed in [29,30]). Of particular interest, a role for the hippocampus in the acquisition and maintenance of MA dependence has been established [31,32]. Related to this recent studies conducted in our laboratory have established that several angiotensin IV (AngIV) analogues possess the ability to bind to and inhibit the activation of hepatocyte growth factor (HGF) and subsequently its receptor c-Met [33,34,35]. Concomitant with this finding several reports have highlighted a potential role for the HGF/c-Met system in cognition [36,37,38,39]. Together these studies prompted us to test whether Divalinal-AngIV (divalinal) could block HGF-dependent activation of c-Met and the acquisition of MA dependence. The results confirmed an important role for the brain HGF/c-Met system in the acquisition of MA dependence. 

## 2. Objectives and Hypotheses

The present investigation utilized an AngIV analogue, divalinal, in an effort to disrupt the acquisition of MA dependence in rats as measured by the conditioned place preference (CPP) paradigm. We hypothesized that the intracerebroventricular (icv) infusion of divalinal prior to exposure to MA during a 5-day acquisition period would reduce drug impact by interfering with hippocampal memory consolidation. We further predicted that following the establishment of MA dependence the icv infusion of divalinal during extinction trials would disrupt subsequent reinstatement.

## 3. Material and Methods

### 3.1. Animals

Male Sprague-Dawley rats (300–350 g, breeding stock derived from Taconic, Germantown, NY, USA) were adapted to a 12 h light/dark cycle initiated at 06:00 h in an American Association for the Accreditation of Laboratory Animal Care-approved vivarium at a temperature of 21 ± 1 °C. The animals were housed individually and provided water and food (Harlan Teklad F6 Rodent Diet, Madison, WI, USA) *ad libitum*, except the night prior to surgery when food was withheld. The protocols utilized in this investigation minimized pain and discomfort, were approved by the Washington State University Institutional Animal Care and Use Committee, and conformed to the guidelines as required by the National Institutes of Health Guide for the Care and Use of Laboratory Animals (NIH Publication No. 80-23). 

### 3.2. Surgery

#### 3.2.1. Chronic Divalinal Infusion

Animals were randomly assigned to four groups (8 rats each). Members of Groups 1 and 2 were each prepared with a mini-osmotic pump (Model 2002 14-day, Alza Scientific Products, Palo Alto, CA, USA) that infused icv at the rate of 0.5 µL/h. Icv delivery was accomplished via a stereotaxically positioned length of hypodermic stainless steel tubing (3.2 cm length of 23 gauge prepared with a 90° bend such that a 7 mm length of tubing was inserted through a skull trephine hole, thus penetrating the roof of the right lateral ventricle while under ketamine-xylazine anesthesia (100 and 2 mg/kg, Phoenix Pharmaceuticals, St. Joseph, MO, USA and Mobay Corporation, Shawnee, KS, USA respectively, i.m.). Flat-skull coordinates for cannula placement relative to bregma were: posterior: 1.0 mm, lateral: 1.5 mm from midline [40]. The cannula was anchored to the cranium with stainless steel screws and dental acrylic. The pump was placed subcutaneously between the scapulae and was connected to the stainless steel tubing via PE-60 tubing (Clay Adams, Parsippany, NJ, USA). Members of Groups 3 and 4 were not prepared with osmotic pumps. We utilized icv infusion given previous results from our laboratory indicating that this route of administration maximally impacted the hippocampus and piriform cortices [41].

#### 3.2.2. Acute Divalinal Infusion

Rats were randomly assigned to four groups (8 rats each) with members of Groups 1 and 2 each prepared with an icv guide cannula (stereotaxic coordinates as indicated above) constructed from PE-60 tubing with a heat bulge that rested on top of the cranium thus serving as a stop to further penetration. The total length of the guide was 2.5 cm with a distance from the beveled tip to the heat bulge of 2.5 mm. Injections were made using a 30-gauge stainless-steel tubing injector with a beveled tip that protruded 2.0 mm beyond the end of the guide cannula. The injector was attached to a 10 µL Hamilton syringe by PE-20 tubing. Members of Groups 3 and 4 were not prepared with icv guide cannulas. 

#### 3.2.3. MA Reinstatement

One group of six rats was used in this experiment. Each animal was prepared with an icv guide cannula as described above. The purpose of this experiment was to first establish MA dependence, and then treat with icv divalinal coupled with extinction trials in an attempt to weaken the association of MA with the nonpreferred CPP compartment.

Correct placement of the guide cannulas used in these experiments was confirmed by the icv injection of 10 µL of fast green dye at the conclusion of testing under Equithesin anesthesia (3.5 mL/kg, i.p., pentobarbital 100 mg/mL; Jensen-Salsbury Laboratory, Kansas City, MO, USA), followed by brain extraction and visual confirmation of dye within the brain ventricles. All osmotic pumps were checked at the completion of the experiment to see whether the contents were exhausted, and they were.

### 3.3. Apparatus

The conditioned place preference apparatus consisted of a conditioning box (length: 64 cm × width: 21 cm × height: 34 cm) with two compartments (each 28 × 21 cm) and a short connecting run (8 × 21 cm). The black compartment had a wire-mesh floor, while the white compartments had parallel metal rods (dia. = 4.8 mm). Each compartment could be isolated by the placement of a white plastic divider door at the entrance. A 15-watt light bulb was positioned above the black compartment in order to decrease the tendency for the animals to spend the majority of their time in the black compartment. A video camera was placed directly over the apparatus to record the activity of the rat. The camera was connected to a computer which recorded the activity interpreted by video tracking software that provided quantifiable information on locomotor activity and time spent in each compartment.

### 3.4. Behavioral Training

#### 3.4.1. Experiment 1: Chronic Divalinal Infusion

**Figure 1:**
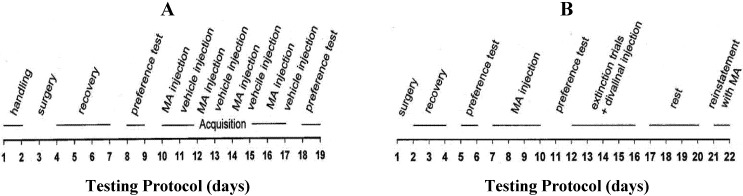
(**A**) Testing protocol for Experiments 1 and 2. Surgery consisted of stereotaxic placement of a chronic icv cannula connected to a mini-osmotic pump filled with divalinal or aCSF (Experiment 1) or the placement of an icv cannula that permitted acute infusion of divalinal or aCSF (Experiment 2). The preference tests were 30 min in duration during which the animal was permitted free access to both compartments. Following MA injection the animal was placed in the nonpreferred compartment for 30 min. Following vehicle injection (0.15 M NaCl, s.c.) the animal was placed in the preferred compartment. (**B**) Testing protocol for Experiment 3. Surgery involved the placement of an icv cannula that permitted acute infusion of divalinal or aCSF. Preference tests were 30 min in duration during which the animal was permitted free access to both compartments. During the drug acquisition phase each animal was given an injection of MA and 5-min later placed in the nonpreferred compartment for 30 min each day. During extinction trials the animal received an icv infusion of divalinal and was placed back into the nonpreferred compartment without MA injection. The rest period was spent in the animal’s home cage. During reinstatement the animals received an MA injection and 5-min later was given free access to both compartments for 30 min.

A 19-day conditioning and testing protocol was used (Figure 1A). Initially the animals were handled for 5-min per day for two days, and then on day 3 members of Groups 1 and 2 were prepared with mini-osmotic pumps. The animals of Group 1 were implanted with pumps that contained divalinal (10 nmol/0.5 µL/h) prepared in artificial cerebrospinal fluid (aCSF). This dose of divalinal is effective at disrupting the acquisition of the Morris water maze task of spatial memory in rats [42]. The pumps used with the animals of Group 2 contained only aCSF and also delivered at a rate of 0.5 µL/h. During four days of recovery handling was continued (5-min per day). The animals were tested for pre-acquisition compartment preference. This was accomplished by permitting each animal to move freely within the CPP box for 30-min on each of two days. Day 8 served to adapt the animal to the apparatus; while the time spent in each compartment on day 9 was utilized to determine compartment preference. On days 10, 12, 14 and 16 each animal received an MA injection (methamphetamine hydrochloride, M8750, Sigma-Aldrich, St. Louis, MO; 2 mg/kg in a volume of 1 mL/kg of sterile 0.15 M NaCl s.c.) 5-min before being confined to the non-preferred compartment for 30 min. On days 11, 13, 15 and 17 these animals received 0.15 M NaCl injection (1 mL/kg s.c.) 5-min prior to being confined to the preferred compartment. On days 18 and 19 each animal was tested for post-conditioning compartment preference for 30 min. Members of Group 3 were not prepared with mini-osmotic pumps but served as drug controls and received MA (2 mg/kg s.c.) according to the above schedule. Members of Group 4 (vehicle controls) were treated identically as the rats of Group 3 but received only sterile 0.15 M NaCl (1 mL/kg s.c.). 

#### 3.4.2. Experiment 2: Acute Divalinal Infusion

The testing protocol for this experiment was generally the same as Experiment 1; however on day 3 the animals of Groups 1 and 2 were each prepared with an icv guide cannula rather than an osmotic pump. Following four days of recovery the animals were tested for compartment preference on days 8 and 9. On days 10, 12, 14, and 16 the animals of Group 1 received MA (2 mg/kg s.c. in 0.15 M NaCl 1 mL/kg) immediately followed by an icv injection of divalinal (10 nmol in 2 µL aCSF) delivered by hand over a 30-s period, 5-min prior to confinement in the non-preferred compartment for 30-min. On days 11, 13, 15, and 17 these rats were subcutaneously injected with saline and icv infused with divalinal and confined to the preferred compartment for 30-min. The animals of Group 2 were treated similarly, however they received MA (2 mg/kg s.c.) immediately followed by an icv injection of aCSF (2 µL) on days 10, 12, 14, and 16 and were confined to the non-preferred compartment for 30-min. On alternate days they received subcutaneous injections of saline followed by an icv infusion of aCSF and were confined to the preferred compartment. Members of Group 3 were not prepared with icv guide cannulas but served as a drug control group as described in Experiment 1 and were taken through the alternating MA and saline vehicle injection protocol as previously described. Members of Group 4 (vehicle controls) were treated identically but received 0.15 M NaCl (1 mL/kg s.c.) rather than MA. On days 18 and 19 each rat was permitted access to both compartments during a 30 min preference test. 

#### 3.4.3. Experiment 3: MA Reinstatement

Rats assigned to this experiment were gentled by handling for 2 days (5-min per day) and were then prepared with an icv guide cannula (day 1, Figure 1B). They were allowed to recover for three days with continued handling each day. On days 5 and 6 compartment preference was determined during a 30 min access to both compartments. During days 7–10 each rat received MA (2 mg/kg s.c.) 5-min prior to confinement in the non-preferred compartment for 30 min. On Day 11 each animal was permitted access to both compartments for 30 min in order to determine preference. On days 12–16 each rat received an icv injection of divalinal (10 nmol in 2 µL aCSF) but no MA 5-min prior to access to both compartments for 30 min. On days 17–20 the rats remained in their home cages. Each rat was then tested for MA-induced reinstatement on days 21 and 22 when they received MA (2 mg/kg s.c.) 5-min prior to being permitted free access to both compartments for 30 min.

### 3.5. Phospho-Met Western Blots

Human embryonic kidney cells 293 (HEK293), were cultured in DMEM, 10% fetal bovine serum (FBS) in 6 well tissue culture plates and grown to 95% confluency. Cells were serum deprived for 24 h prior to treatment to reduce the basal levels of phospho-Met. Following serum starvation, cocktails comprised of vehicle, HGF with/without divalinal at 10^−12^, 10^−10^, and 10^−8^ M were prepared and pre-incubated for 30 min at room temperature. The cocktail was then added to the cells for 10 min to stimulate the c-Met receptor and downstream proteins. The cells were lysed in ice-cold Ripa buffer (Millipore, Billerica, MA, USA) with protease and phosphatase inhibitors (Sigma-Aldrich; St. Louis, MO, USA). The lysate was clarified by centrifugation at 15,000× *g* for 15 min, protein concentrations were determined using the BCA total protein assay (Pierce Chemical; Rockford, IL, USA), and then appropriate volumes of the lysates were diluted with 2× reducing Laemmli buffer and heated for 10 min at 95 °C. 20 µL of lysate. These were resolved using SDS-PAGE (Criterion, BioRad Laboratories; Hercules, CA, USA), transferred to nitrocellulose, and blocked in Tris-buffered saline (TBS) containing 5% milk for 1 h at room temperature. The phospho-Met antibody (ab5662, Abcam, Cambridge, MA, USA) was added to the blocking buffer at a final concentration of 1:1000 and incubated at 4 °C overnight with gentle agitation. Membranes were then washed several times with TBS, a 1:5000 dilution of horseradish-peroxidase conjugated goat anti-rabbit (Pierce Chemical; Rockford, IL, USA) was added, and the membranes further incubated for 1 h at room temperature. The membranes were washed several times with TBS before being developed by chemiluminescence (Pierce Chemical; Rockford, IL, USA), and the bands detected and quantitated using a UVP phosphoimager (Upland, CA, USA).

### 3.6. Scattering Assay

Madin-Darby Canine Kidney (MDCK) cells were grown to 100% confluency on coverslips in six-well plates and washed twice with PBS. The confluent coverslips were then aseptically transferred to new six well plates containing 900 µL serum free DMEM. Divalinal at 10^−14^, 10^−12^, 10^−10^, 10^−8^ M, and/or HGF (20 ng/mL) were added to appropriate wells. Control wells received PBS vehicle. Plates were incubated at 37 °C with 5% CO_2_ for 48 h. Media was removed and cells were fixed with methanol. Cells were stained with Diff-Quik Wright-Giemsa (Dade-Behring, Newark, DE, USA) and digital images were taken. Quantification of images was achieved and statistics were performed using Prism 5 and InStat v.3.05 (GraphPad; San Diego, CA, USA).

### 3.7. Compounds

Methamphetamine was dissolved in sterile 0.15 M NaCl. Artificial cerebrospinal fluid (in mM: 124 NaCl, 3 KCl, 1.24 KH_2_PO_2_, 1.3 MgSO_4_, 2.0 CaCl_2_, 26 NaHCO_3_, and 10 D-glucose) was prepared in aliquots and frozen at −40° until used. Once used the aliquot was discarded. Divalinal-AngIV (Val-ψ-Tyr-Val-ψ-His-Pro-Phe, where ψ = reduced peptide bond CH_2_–NH_2_) was synthesized in our laboratory using an automated peptide synthesizer (Coupler 250, DuPont, Wilmington, DE, USA). The peptide purity of divalinal was 90% with acetate representing the major contributor to the decreased peptide content. Correction was made for peptide purity when the compound was prepared. HGF was purchased from R & D systems (Minneapolis, MN, USA).

### 3.8. Statistical Analysis

Because of the minimal, but variable, amount of time that the rats could spend in the connecting run preference data were converted to percent coefficients according to the formula [43]:

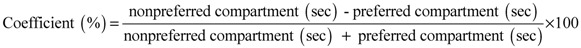


One-way analysis of variance (ANOVA) was used to analyze the data sets of Experiments 1 and 2 regarding pre-and post-acquisition compartment preferences, and the area densities of the Western blots. Significant effects were further analyzed using Newman-Keuls *post-hoc* tests with a level of significance set at *p* < 0.01. Paired *t*-tests were used to compare pre-and post-MA compartment preferences in Experiment 3 with a level of significance set at *p* < 0.01.

## 4. Results

### 4.1. Experiment 1: Chronic Divalinal Infusion

**Figure 2:**
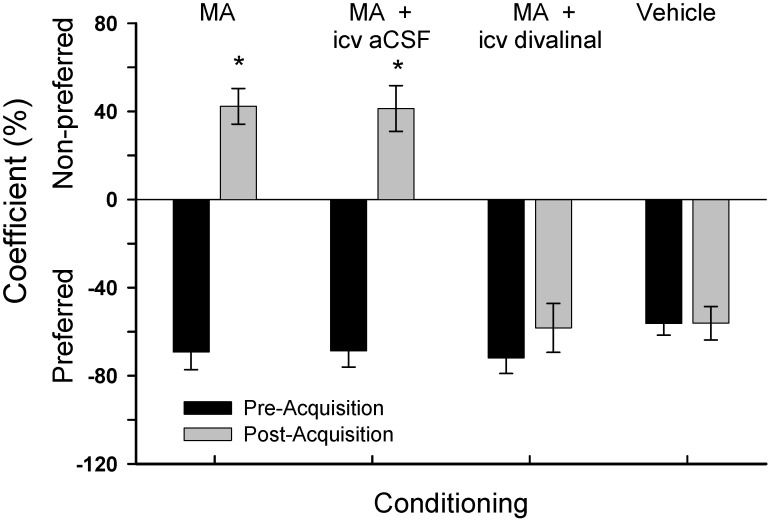
Mean ± SEM percent time spent in the designated compartment (Preferred or Non-preferred) by the four groups in Experiment 1. Following acquisition trials the animals given only MA showed a clear shift from the preferred to the nonpreferred compartment suggesting dependence. This behavioral shift was also seen with those animals injected with MA and icv infused with aCSF via mini-osmotic pumps. Members of the group given MA and icv infused with divalinal failed to show a shift to the nonpreferred compartment suggesting no dependence. As expected those animals given vehicle (s.c.) continued to stay in the preferred compartment. * *p* < 0.01.

The results of this experiment utilizing icv osmotic pump delivery of divalinal or aCSF are presented in Figure 2. There were no differences among the groups regarding the time spent in the preferred compartment during pre-acquisition preference testing (*F*_3,28_ = 0.89, *p* > 0.10). Following the MA and vehicle injection protocol there were group differences in preference (*F*_3,28_ = 39.25, *p* < 0.001). *Post-hoc* analyses indicated that MA treatment resulted in a shift in preference to the non-preferred side by members of the group given only subcutaneous MA, and also for the group given subcutaneous MA combined with the icv infusion of aCSF, indicating drug dependence. In contrast, those animals given MA injection plus icv infused divalinal failed to shift to the non-preferred compartment suggesting no dependence. As expected those animals given only subcutaneous vehicle also failed to shift preference.

### 4.2. Experiment 2: Acute Divalinal Infusion

**Figure 3:**
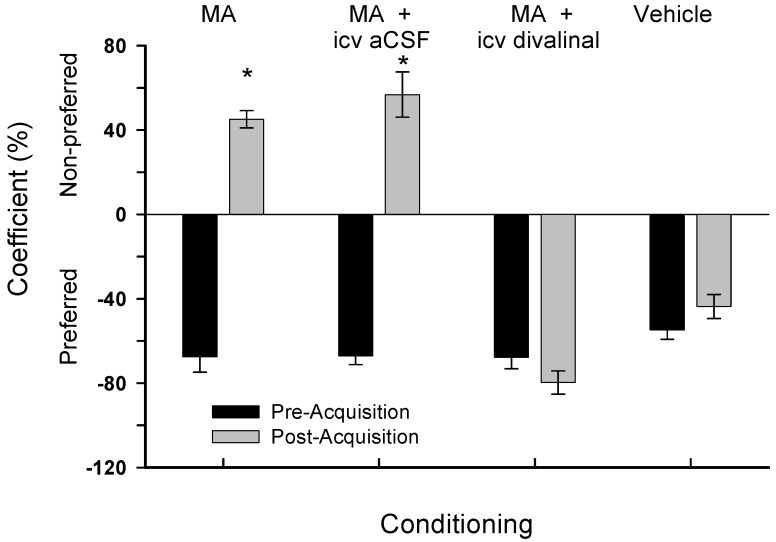
Mean ± SEM percent time spent in the designated compartment by the four groups utilized in Experiment 2. These results are equivalent with those presented in Figure 2. Members of the icv infusion groups were each prepared with an icv cannula and received acute infusions. * *p* < 0.01.

Results from treatment with acute icv infusion of divalinal or aCSF are presented in Figure 3. There were no differences among the groups concerning the time spent in the preferred compartment during pre-acquisition preference testing (*F*_3,28_ = 1.81, *p* > 0.10). Following the acquisition protocol there were group differences in preference (*F*_3,28_ = 57.87, *p* < 0.001). *Post-hoc* analyses revealed that MA treatment shifted preference to the non-preferred compartment for members of the group given only subcutaneous MA, and those animals given subcutaneous MA plus icv aCSF infusion, indicating drug dependence. However, members of the group injected with MA plus icv infused divalinal failed to shift to the non-preferred compartment, suggesting no dependence. As expected those animals given only subcutaneous vehicle also failed to shift compartment preference.

### 4.3. Experiment 3: MA Reinstatement

**Figure 4:**
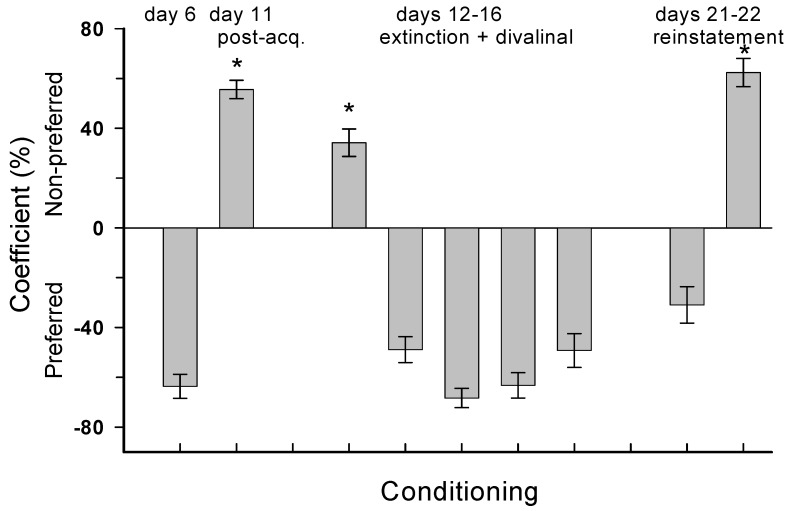
Mean ± SEM percent time spent in the designated compartment. Following acquisition trials to MA (days 7–10) the animals demonstrated a shift from the preferred (day 6) to the nonpreferred (day 11) compartment. During extinction trials (days 12–16) the animals received icv infusions of divalinal 5-min prior to access to both compartments for 30 min on each day and evidenced a shift back to the preferred compartment. On days 17–20 the rats were rested in their home cages. On days 21 and 22 each rat received MA followed 5-min later with access to both compartments. During these two days there was a clear shift back in favor of the nonpreferred compartment indicating reinstatement of dependence. * *p* < 0.01.

Results are presented in Figure 4. There was a significant shift toward drug dependence comparing pre-acquisition (day 6) to post-MA treatment (day 11) compartment preferences (*t*_5_ = 10.66, *p* < 0.001). During extinction trials acute icv infusion of divalinal was administered to each animal and there was a reversal back to the preferred compartment. Following four days of rest these animals experienced reinstatement testing. On Day 21 the animals continued to spend the majority of their time in the preferred compartment but a trend toward the nonpreferred compartment was noted. On day 22 the animals showed a clear shift to the nonpreferred side suggesting MA-induced reinstatement of dependence (*t*_5_ = 8.92, *p* < 0.001).

### 4.4. Divalinal Augments HGF-Dependent Met Signaling and Cellular Activity

**Figure 5:**
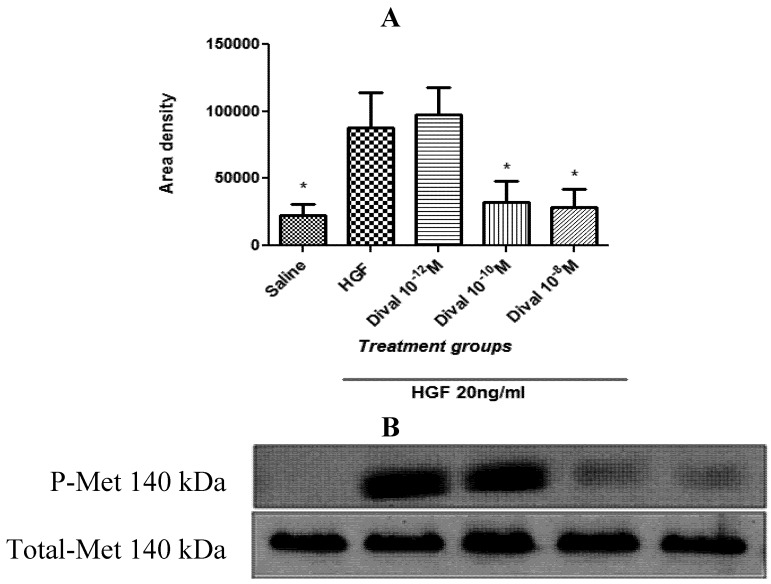
Divalinal inhibits hepatocyte growth factor (HGF)-dependent c-Met activation. (**A**) HEK293 cells were treated for 10 min with HGF ± divalinal at 10^−12^, 10^−10^, or 10^−8^ M. HEK293 cell lysates were immunoblotted with anti-phospho-Met and anti-Met antibodies. Both treatment groups (HGF 20 ng/mL + Dival at 10^−10^ and 10^−8^ M) were statistically different from the HGF treated group (* *p* < 0.05), but were not different from one another or non-treated controls (*p* > 0.05). Mean ± SEM, *N* = 3. (**B**) Western blots for P-Met and total-Met.

Previous studies conducted in our laboratory indicated that other AT_4_ receptor antagonists are potent inhibitors of the HGF/c-Met system [33,34,35], so in the present investigation we determined whether divalinal acts to inhibit c-Met signaling. c-Met is a tyrosine kinase-linked growth factor receptor, thus c-Met activation requires a tyrosine residue auto-phosphorylation step that is essential for the eventual recruitment of various SH2 domain signaling proteins. We first evaluated the ability of divalinal to inhibit c-Met tyrosine phosphorylation. As anticipated, divalinal was an effective blocker of HGF-dependent c-Met phosphorylation at 10^−10^ and 10^−8^ M (* *p* < 0.05; Figure 5) but had no effect on total c-Met expression. The divalinal dose of 10^−12^ M failed to block Met phosphorylation.

### 4.5. Scattering Assay

**Figure 6:**
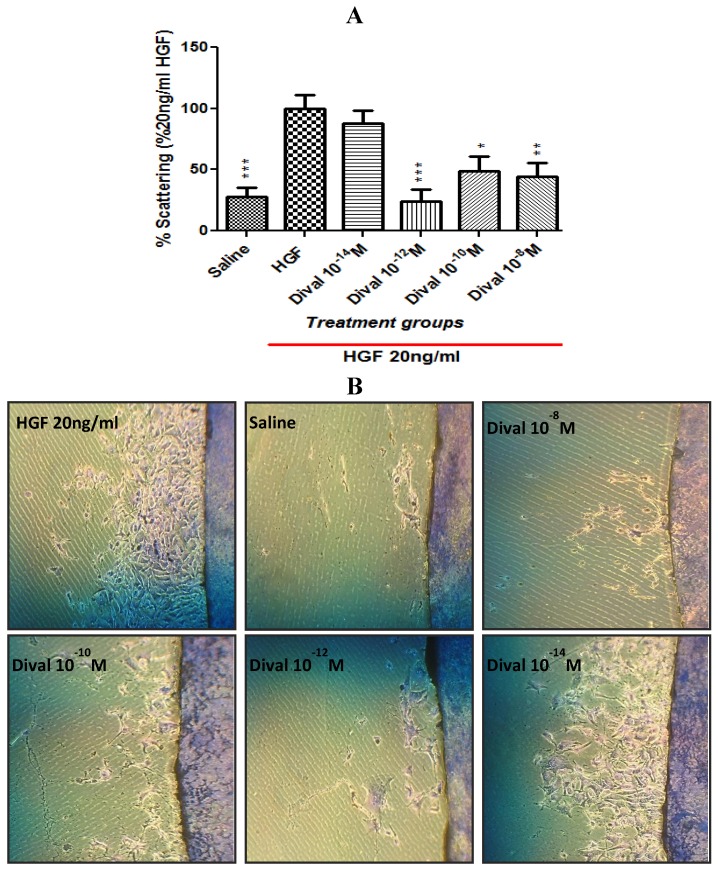
Divalinal inhibits HGF-dependent scattering in MDCK cells. Cell scattering in which cells lose cell-to-cell contact and then migrate is the classic response to HGF. MDCK cells are the gold standard cellular model for studying the HGF/Met system. These cells were grown to 100% confluence on coverslips and then placed on a clean plate. The cells were stimulated to scatter off of the coverslip by adding 20 ng/mL of HGF to the media alone or in combination with divalinal at 10^−14^,10^−12^,10^−10^ or 10^−8^ M. After 48 h of scattering, the cells were fixed with methanol and stained with Diff-Quik. The coverslips were removed to reveal the ring of cells that had scattered off the cover slip and onto the plate. (**A**) Quantification of HGF effect on cell scattering. Treatment groups (10^−12^, 10^−10^and 10^−8^ M) were statistically different than the HGF treated group (* *p* < 0.05, ** *p* < 0.01, *** *p* < 0.001), but not different from one another or non-treated controls (*p* > 0.05). Mean ± SEM, *N* = 8. (**B**) Pictures of MDCK cells scattering off their coverslips.

Divalinal’s capacity to block HGF-dependent Met phosphorylation predicts that it should inhibit HGF-dependent cellular processes including proliferation, migration, invasion, and relief from anoikis (blockade of apoptosis). In this regard we chose to evaluate the effect of divalinal on HGF-dependent cell scattering, which is a hallmark cellular response to c-Met activation by HGF [44]. This response relies on the loss of cellular adhesion and increased motogenic activity. HGF-dependent scattering was assessed in Madin-Darby Canine Kidney (MDCK) cells, a standard cellular model for investigating the HGF/Met system [45], and well recognized for its robust scattering response to HGF. MDCK cells grown at a low cell density form colonies and evidence a “cobblestone” morphology, characterized by tight intercellular junctions. Application of HGF initiates a scattering response that occurs in two stages [46]. First, the cells lose their cell-to-cell adhesion and become polarized. Second, they separate and migrate away from each other. If divalinal behaves similarly with other AT_4_ receptor antagonists it would be expected to attenuate scattering in MDCK cells stimulated with HGF, thus disrupting a prominent HGF/Met initiated cellular activity. This expectation was verified (Figure 6). These data illustrate that divalinal attenuates MDCK cell scattering. A maximum divalinal effect was observed at 10^−10^ and 10^−8^ M, with a threshold near 10^−12^ M.

## 5. Discussion

The development of drug addiction is in part dependent upon neural plasticity events that provoke alterations in synaptic morphology and the distribution of synaptic proteins reminiscent of the synaptic changes accompanying normal learning and memory consolidation (reviewed in [19,24,26,47,48,49,50]). The realization that the addictive process requires the engagement of plasticity events and changes in synaptic proteins typically associated with cognition offers a potential treatment strategy for drug dependence, namely the use of pharmaceuticals that target proteins critical to learning and memory consolidation. Despite the relative infancy of this approach a number of studies have validated its potential utility. For example, MMPs are responsible for the physical synaptic remodeling that is essential for spatial learning and memory consolidation [51]. MMP disruption results in impaired reinstatement of cocaine-induced self-administration and CPP [9,10,52]. Intracerebroventricular infusion of a broad spectrum MMP inhibitor also attenuates escalated ethanol self-administration following chronic or acute exposure to ethanol [53]. The inhibition of ERK activity, which plays an important role in learning [54], has been shown to block cocaine-induced adaptive behaviors associated with addiction. And the NMDA blocker, K801, disrupts cocaine-dependent CPP reinstatement [55]. Based on this functional linkage of addiction to synaptic plasticity we hypothesized that blockade of hippocampal synaptogenesis of the brain AngIV/AT_4_ receptor system [56] would interfere with MA-dependent CPP.

The notion of using an AT_4_ receptor antagonist to interfere with addiction evolved from previous work in this research area. Braszko and colleagues [57,58] were first to report that icv injected AngIV facilitated exploratory behavior by rats tested in an open field, and improved both recall of passive avoidance conditioning and the acquisition of active avoidance conditioning. Our laboratory confirmed and extended these findings in that icv infused AngIV was shown to facilitate the recall of a passive avoidance response in a dose-dependent fashion [59]. Further, icv infused metabolically resistant analogues of AngIV facilitated acquisition of the Morris water maze task; while animals icv infused with aCSF, or an inactive pentapeptide that did not bind at the AT_4_ receptor, were much less successful [42]. A similar facilitation of spatial memory acquisition by AngIV analogues was observed in rats made amnesic with the cholinergic muscarinic receptor antagonist scopolamine [60], and hippocampal perforant path knife-cut rats [42]. The fact that icv infusion of divalinal disrupted recall of the passive avoidance response, and also significantly inhibited the acquisition of the Morris water maze task [42], suggested that this AT_4_ receptor antagonist may be useful in disrupting drug-induced memory consolidation and led to the present investigation. These findings also raise the important concern that clinical use of such an antagonist could negatively impact all learning and memory. This issue deserves further attention. One possible experimental approach is to utilize the four group protocol of Experiment 2 and test these animals on additional tasks designed to measure the acquisition of spatial memory (e.g., Morris water maze), associative memory (e.g., passive avoidance conditioning), and novel object recognition. In this way it may be possible to determine the impact of divalinal on other types of memory formation.

Present findings provide support for the involvement of the Ang IV/AT_4_ receptor system in MA dependence. The major findings are: (1) Continuous icv infusion of divalinal via mini-osmotic pump prevented the acquisition of MA-induced CPP. (2) A similar disruption of the acquisition of MA-induced CPP was seen with acute icv infusion of divalinal. (3) Divalinal impaired memory reconsolidation of the MA-associated environmental context cues during extinction. However, (4) acute icv infusion of divalinal during extinction trials failed to suppress MA-primed reinstatement of CPP. It is noteworthy that acute injection of divalinal was as efficacious as chronic infusion at reducing the rewarding effects of MA, and both discouraged acquisition of MA dependence. However, these results indicate that the AngIV/AT_4_ receptor system is not critical to memory reconsolidation associated with reinstatement [19,61]. On the other hand, this could mean that the experimental design failed to temporally coordinate the presence of divalinal with the reconsolidation event. Future studies will explore this question by pairing divalinal exclusively with the MA-associated side of the chamber without intervening extinction plus or minus the priming dose of MA. We will also examine the acute dosing regimen with regard to frequency.

Although not tested directly *in vivo* in this study, results presented here suggest that divalinal’s biological actions are dependent on inhibition of the HGF/c-Met system. By inference acquisition of MA-dependent CPP is likewise reliant on a functioning brain HGF/c-Met system. Supporting data demonstrated that divalinal at 10^−8^ and 10^−10^ M, but not 10^−12^ M, effectively blocked HGF-dependent phosphorylation/activation of c-Met. Further, divalinal at the above mentioned concentrations was capable of dramatically suppressing HGF-dependent cell scattering in MDCK cells, the gold standard method for visualizing and monitoring HGF/c-Met-dependent cellular activity. The inability of 10^−12^ M divalinal to attenuate c-Met phosphorylation was most likely the result of very short incubation times that were insufficient for the complete association of such a low concentration of divalinal with HGF. Divalinal is an exceptionally stable compound [62], and when continuously present throughout the entire 48 h scattering experiment was capable of blocking MDCK cell scattering. 

Recent studies from our laboratory indicate that classic AT_4_ receptor antagonists are potent inhibitors of c-Met activation and act as direct allosteric blockers of HGF dimerization and subsequent activation [33,34,35]. As such our expectation was that divalinal, similar with other AT_4_ receptor antagonists, would block HGF dimerization and activation. Present results support the hypothesis that the molecular target of AngIV analogues is HGF, with the c-Met receptor as the ultimate functional target.

It is presently not known whether all of the cellular effects of brain delivered AngIV analogues can be attributed to modulation of the HGF/c-Met system; however there is a growing literature linking HGF to learning, synaptic plasticity, neuroprotection, and neurogenesis. Similar to AngIV and AngIV analogues, HGF has been shown to augment learning [36,38,39]. Consistent with a role in cognition c-Met is localized at glutamatergic synapses in the hippocampus where it is found concentrated at the postsynaptic density [63]. Predictably, HGF [36] like AngIV and AngIV analogues [64,65], facilitates long-term potentiation (LTP) at CA1 synapses in the hippocampus. A common correlate to LTP is an expansion of the communicating dendritic network accompanied by an increase in the number and size of dendritic spines. In accord with this expectation both AngIV analogues [56] and HGF [66,67] initiate changes in dendritic morphology. In addition to its impact on synaptic function the HGF/c-Met system plays a critical role in neural development and exhibits general neurotrophic/neuroprotective/neurogenic properties [68,69,70,71]. It is also possible that divalinal disrupts insulin-regulated aminopeptidase (IRAP) and related cellular glucose uptake (reviewed in [72]). These possibilities must be addressed in future studies.

The action of divalinal on two major neurotransmitter systems connected to addiction, the cholinergic and dopaminergic systems, may provide a potential mechanism for its ability to blunt the development of CPP. Although there is presently no link between the brain HGF/c-Met and cholinergic systems, linkage to AngIV has been established. Lee [73] reported that AngIV stimulates acetylcholine release from the hippocampus. Further, Norleucine^1^-AngIV delivered icv is capable of reversing the amnesia caused by the muscarinic antagonist scopolamine [42]. The brain cholinergic system has long been of interest to researchers studying cognitive function (reviewed in [74,75]) and addiction (reviewed in [76]). Particularly relevant is the contributory role of cholinergic mechanisms to MA-induced behaviors [77,78], and the ability of the cholinergic agonist nicotine to act synergistically with psychostimulant drugs to increase DA levels in the nucleus accumbens [79,80]. It remains to be determined whether inactivation of the brain HGF/c-Met system, or the actions of AT_4_ receptor antagonists on MA-dependent CPP, are reliant on alterations of the cholinergic system.

A second potential neurotransmitter target of divalinal is the brain dopaminergic (DA) system, known to be intimately associated with reward and drug addiction. Braszko [81] has convincingly shown that both acetylcholine and DA are critical participants in the enhanced memory consolidation apparent following application of an AngIV analogue. In support of a potential functional bond between the Ang IV and DA systems, AngIV has been shown to facilitate DA release in the striatum [82,83]. In addition to a connection between DA and AngIV there appears to be a linkage between DA and the HGF/c-Met system. Hamanoue and colleagues [84] have reported that HGF can augment the development of tyrosine hydroxylase-positive neurons in rat mesencephalic cultures as well as DA uptake into these mesencephalic neurons. Thus, an alternative or additional explanation for divalinal’s actions on CPP could be decreased striatal DA release and consequent reduced reward mediated through HGF/c-Met system blockade. 

## 6. Conclusion

The present results confirmed the hypothesis that an AT_4_ receptor antagonist, divalinal, is capable of disrupting the memory of environmental retrieval cues associated with the acquisition of MA-dependent CPP. Further, these data support the identification of the AT_4_ receptor subtype as HGF and suggest a potentially critical role for the brain HGF/c-Met receptor system in mediating at least the initial acquisition of MA dependence. These findings encourage the examination of blood-brain permeable HGF/c-Met antagonists for the treatment of drug dependence [85]. However, it will be necessary to determine whether the use of an AT_4_ receptor antagonist negatively impacts other types of memory in addition to that linked with drug dependence. Specific details concerning the neurotransmitter related mechanism of action as related to divalinal, and the neuroanatomical location of these influences, remain to be determined.
